# Effect of Energy and Temperature on Tetrahedral Amorphous Carbon Coatings Deposited by Filtered Laser-Arc

**DOI:** 10.3390/ma14092176

**Published:** 2021-04-23

**Authors:** Frank Kaulfuss, Volker Weihnacht, Martin Zawischa, Lars Lorenz, Stefan Makowski, Falko Hofmann, Andreas Leson

**Affiliations:** 1 Fraunhofer Institute for Material and Beam Technology (IWS), 01277 Dresden, Germany; volker.weihnacht@iws.fraunhofer.de (V.W.); martin.zawischa@iws.fraunhofer.de (M.Z.); stefan.makowski@iws.fraunhofer.de (S.M.); falko.hofmann@iws.fraunhofer.de (F.H.); andreas.leson@iws.fraunhofer.de (A.L.); 2 Institute of Manufacturing Science and Engineering, Technische Universität Dresden, 01069 Dresden, Germany; lars.lorenz@iws.fraunhofer.de

**Keywords:** diamond-like carbon (DLC), tetrahedral amorphous carbon (ta-C), coating, superhardness, friction, wear, laser-arc

## Abstract

In this study, both the plasma process of filtered laser-arc evaporation and the resulting properties of tetrahedral amorphous carbon coatings are investigated. The energy distribution of the plasma species and the arc spot dynamics during the arc evaporation are described. Different ta-C coatings are synthesized by varying the bias pulse time and temperature during deposition. An increase in hardness was observed with the increased overlapping of the bias and arc pulse times. External heating resulted in a significant loss of hardness. A strong discrepancy between the in-plane properties and the properties in the film normal direction was detected specifically for a medium temperature of 120 °C during deposition. Investigations using electron microscopy revealed that this strong anisotropy can be explained by the formation of nanocrystalline graphite areas and their orientation toward the film’s normal direction. This novel coating type differs from standard amorphous a-C and ta-C coatings and offers new possibilities for superior mechanical behavior due to its combination of a high hardness and low in-plane Young’s Modulus.

## 1. Introduction

The development of low-friction surface materials is important for improving engine efficiency and reducing CO_2_ emissions. Diamond-like carbon (DLC) is a material of specific interest because it combines the properties of high hardness and low friction. Consequently, various forms of DLC have been studied as resilient, low-friction surface coatings. DLC describes a whole class of coating variants consisting of a proportion of sp^2^- and sp^3^-hybridized carbon atoms and, depending on the manufacturing process, a fairly large proportion of hydrogen. The systematic classification of the different variants of carbon-based coatings can be found in the ISO standard 20523:2017 [1]. Depending on the composition, bonding state, and nanostructure, such coatings cover a broad range of different properties [2].

At present, DLC type a-C:H is the most widespread in industry. It is typically produced in plasma-assisted CVD or reactive sputter deposited processes by the decomposition of hydrocarbons. Therefore, these layers contain a comparatively high proportion of hydrogen and typically show a moderate hardness.

In recent years, hydrogen-free tetrahedral amorphous carbon (ta-C) coatings have increasingly become part of industrial production. This is because ta-C coatings have several advantages over established DLC coatings due to their superhardness (>40 GPa), making them extremely wear-resistant. Furthermore, in combination with special lubricants, they can show an extraordinarily low friction, so-called superlubricity [3,4].

For the production of ta-C coatings, various PVD processes can be considered. However, for the atomistic formation process of sp^3^ bonds in ta-C, the impinging carbon atoms or ions require a certain energy range of around 100 eV. Therefore, PVD processes with low particle energies or low ionization (e.g., e-beam evaporation or conventional sputtering) are not suitable for ta-C synthesis. The cathodic arc process, on the other hand, has been established for ta-C manufacturing because it not only provides plasma in a suitable energy range but also has a high deposition rate [5]. A process-related disadvantage of the cathodic arc is the fact that, in addition to the plasma, a large number of macro-particles are generated, which leads to a relatively rough surface. By deflecting the plasma using electromagnetic fields, however, these particles can be separated from the plasma [6].

In contrast to arc processes on metal cathodes, as used for the production of nitride hard coatings, the arc spot on graphite cathodes is difficult to control. This problem can be fundamentally circumvented with a laser-triggered arc ignition. The basic principle of the laser-induced vacuum arc evaporation (laser arc) process has been described in detail by Scheibe and Siemroth [7,8,9], and consequently developed to industrial maturity at the Fraunhofer IWS. This concept was later extended by the integration of a plasma filter [10]. The plasma filter deflects the coating plasma at 60° with the help of electric and magnetic fields, which results in a separation of the plasma from the emitted particles and, hence, in ta-C coatings with a significantly improved surface quality.

It is well known that the properties of ta-C coating depend, to a large extent, on the plasma parameters. By varying the bias voltage, the effective particle energy and, thus, the sp^3^ content can be adjusted [11,12]. An increased temperature during deposition leads to relaxation phenomena and thus has a decisive influence on the sp^3^/sp^2^ ratio and the coating properties [13]. Schultrich theoretically investigated the influence of energy and temperature in detail [14]. While a number of experimental studies were conducted for ta-C coatings deposited by conventional DC arc processes [12,15,16], no such results are available for pulsed processes, especially the laser-arc process. Therefore, the aim of this paper is to investigate the influence of bias voltage and temperature on the coating properties specifically for the laser-arc process. First, fundamental studies were carried out on pulsed laser-arc plasma to measure the energy distribution of carbon species. Due to the nature of the pulsed method, it was also necessary to consider the time-related aspects of arc-spot dynamics.

## 2. Experiments 

### 2.1. Description of Filtered Laser-Arc Deposition Technique 

**Figure 1:**
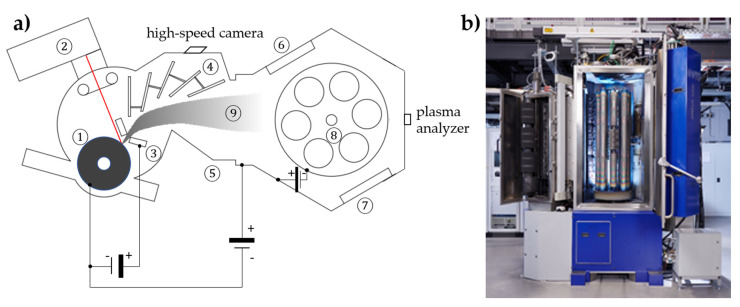
(**a**) Scheme of the coating systems with (1) graphite cathode, (2) laser scanner, (3) anode, (4) filter, (5) chamber/ground, (6) radiation heater, (7) magnetron, (8) substrate rotation, and (9) carbon plasma. (**b**) View of the coating system.

The experimental setup is shown in Figure 1. The evaporation was carried out in a high vacuum at a pressure of about 10^−4^ Pa. The plasma was produced by the pulsed vacuum arc erosion of the graphite cathode (target). The repetitive ignition of the arc pulses was triggered by a pulsed laser using a commercial Q-switched Nd-doped yttrium aluminum garnet (Nd-YAG) laser. The laser pulse length was about 100 ns, with an energy of about 15 mJ, which is smaller than the arc-discharge energy by a factor of 1000 (approx. 15 J). The combination of the linear scanning of the laser spot and rotation of the graphite target results in uniform erosion and homogeneous plasma distribution. The employed current source provides a sinusoidal current up to 1600 A at a discharge duration of 320 µs for the coating process at a repetition rate of up to 900 Hz. To allow low coating temperatures for these studies, the maximum current is limited to 1450 A and the repetition rate is set to 300 Hz. The substrate is set in two-fold rotation and is equipped with a bias voltage source, which allows defined pulse overlaps. The deposition rate is about 0.24 µm/h at this repetition rate. For measuring the ionization states and energy distribution of carbon species, the plasma analyzer Hiden EQP (Hiden Analytical, Warrington, UK) was used. It was placed in the rear part of the coating chamber after removing the substrate holder table. High-speed images were taken with a Fastcam SA-X2 from Photron (West Wycombe, UK) with a frame rate of 100,000 fps. The filter setup was removed to gain an unhindered view of the arc spot.

### 2.2. Materials, Parameters, and Test Methods 

The main objective of this investigation was to study the influence of carbon ion energy and deposition temperature on the properties of ta-C coatings deposited by the filtered laser-arc technique. The ta-C coatings were deposited on steel samples and subsequently characterized with respect to their structural features and mechanical properties.

Hardened low-alloy chromium steel (100Cr6, EN 1.3505, SAE 52100) was used as a substrate for all coatings. Flat samples of 18 mm × 13 mm × 3 mm were polished to Ra < 20 nm. Prior to deposition, the samples were cleaned in an ultrasonic bath with an alkaline solution and then dried. For coating, they were fixed on a holder in an 8-axis planetary system and coated in a twofold rotation. The coating process started with an argon ion etching step using a hollow cathode. Next, a 100 nm-thick Cr adhesion layer was deposited by magnetron sputtering followed by ta-C deposition using filtered laser-arc.

In this work, the following variations (Table 1) were made to alter the properties of ta-C coatings during laser-arc evaporation at a maximum arc current of 1450 A:

1. Variation in bias characteristics. A pulsed bias voltage of 100 V was applied synchronously to the arc pulses. The duration of the bias pulse was set to 175 and 250 µs. For comparison, one coating was deposited with a 0 V bias voltage.
2. Application of external heating during ta-C deposition using a radiant heater, which was positioned on the wall of a coating chamber. The temperature sensor used to control the temperature was on the chamber wall near the substrate holder. The temperature was set to 120 or 140 °C.

The parameters for coating B were established standard Laser-arc parameters used for numerous industrial deposition processes before. Therefore, 100 V and 175 µs were chosen as bias voltage and bias pulse duration, respectively, for the temperature variation series (II).

All ta-C coated steel samples were characterized using the following techniques. The Young’s modulus of the coating was quantified by laser-induced surface acoustic wave spectroscopy (LAwave) [17]. Additionally, the hardness of coatings was measured with the ZHN (Zwick/Roell, Ulm, Germany) nanomechanical hardness tester. A Berkovich indenter was loaded to a maximum force of up to 100 mN using the quasi continuous stiffness method (QCSM) and evaluated according to ISO 14577-4 [18]. At least 10 indentations were performed on each sample. Before the measurement, the surfaces were smoothed in order to reduce the scatter of the hardness results due to the high surface roughness. Coating thickness was measured by the ball crater-grinding method and confirmed by LAwave, which yielded the film thickness as well.

A Renishaw inVia Raman microscope (Wotton-under-Edge, UK) with a 50× objective and an excitation wavelength of 514 nm was used for Raman spectroscopy. The laser power on the sample was estimated to be 0.8 mW. The spectra were fitted using a linear base line, a Lorentzian line for the D peak, and a Breit–Wigner–Fano line for the G peak [19]. For numerical evaluation, the position of the G peak maximum and the peak height ratio of the D and G peaks were calculated accordingly. At least three Raman spectra were recorded for each coating. The fitting range was adjusted to cut out the signal from the surrounding baseline, usually resulting in a window of 1000 to 1850 cm^−1^.

For investigations with transmission electron microscopy (TEM), we used the JEOL JEM-2100 (Tokyo, Japan) configured with a LaB_6_ cathode, a high-resolution polepiece (HRP), and an AZtec X-Max EDS system. Samples were thinned to electron transparency through a sequence of grinding, polishing, and Ar ion beam thinning in a Precision Ion Polishing System “PIPS695” (Gatan Inc., Pleasanton, CA, USA). 

## 3. Results and Discussion

### 3.1. Fundamentals of Laser-Arc Evaporation

#### 3.1.1. Plasma Investigations

**Figure 2:**
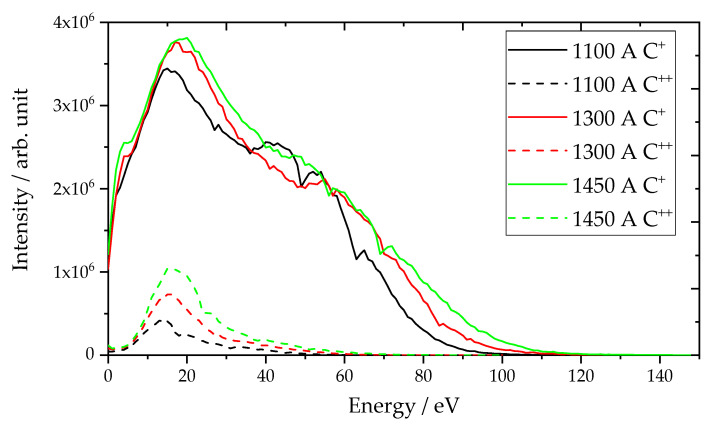
Energy distribution of single- and double-charged carbon ions at 1100–1450 A.

The results of the filtered carbon plasma investigation using a plasma analyzer are presented in Figure 2. The average calculated ion energies are listed in Table 2. Different maximum arc-discharge currents were used to analyze the effect of arc current on the energy distribution of single- (C^+^) and double-charged (C^++^) carbon ions. The curves for both ionization stages show a maximum at low energies of 15 to 19 eV and a pronounced shoulder at higher ion energies, with maximum ion energies of 120 eV at the highest discharge current of 1450 A for single-charged ions. For the double-ionized species, a maximum energy of 70 eV was found at the highest current. Reference [20] describes sp^2^-rich coatings with about 10% sp^3^ content at ion energies of up to 20 eV, followed by a transition from predominately sp^2^ bonded a-C to predominately sp^3^ bonded ta-C between 20 and 30 eV. Regarding the mean energies, ta-C films were formed for all coating parameters.

Similar values for single-charged ions were described in former investigations using laser-arc evaporation [21]. Even in studies [22,23,24] using deposition techniques other than laser-arc, common ion energy distributions of 20–50 eV were reported, which is in line with the average ion energies of around 31 eV determined here. The plasma was found to contain a small proportion of double-charged ions. This is important to mention, as double charge means a double acceleration effect by bias voltage and, therefore, significantly higher particle energies in processes using bias voltage.

The average energy (listed in Table 2) increases from 30.8 to 32.6 eV for single-charged ions and from 16.7 to 19.6 eV for double-charged ions. The proportion of double-charged ions increases with increasing current from 4.3% at 1100 A to 10.4% at 1450 A. A maximum value of 15% is given in [5]. An explanation for the small differences between the average energies can also be the spot splitting as a result of the spot splitting current (see also Section 3.1.2). Each spot should provide a comparable plasma energy distribution, but the number of spots increases the magnetic flux density on the cathode surface and thus the arc spot velocity. The higher velocity lowers the spot temperature and thus increases the average ion energy and fraction of higher excited species [25,26].

#### 3.1.2. Cathode Processes

**Figure 3:**
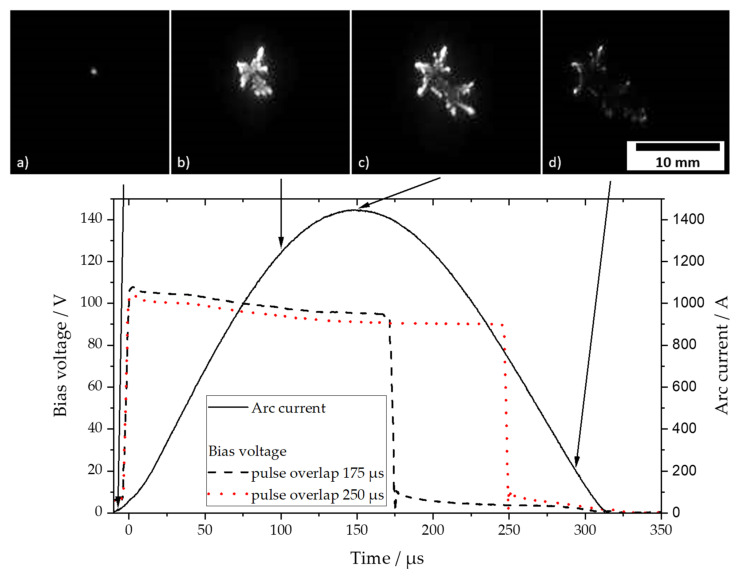
Time-resolved arc current and different bias pulse overlaps and arc-spot images spreading at 1450 A at (**a**) 0 µs (laser ignition), (**b**) 100 µs, (**c**) 150 µs (maximum current), and (**d**) 300 µs.

The sinusoidal shape of the discharge current has an influence on the formation and movement of the arc spots (Figure 3). The discharge is started within a spot by the laser pulse (a). Due to the increasing current, the arc spots branch out more and more (b). Reference [25] describes the causal mechanism as a spot splitting current. For graphite surfaces, 200 A is given. Therefore, when the maximum current is reached, 6 separate areas of the arc spot can be identified (c). With decreasing current, no new arc spots are formed and the spots are gradually extinguished (d). From the spot dynamics, the maximum velocities of about 40 m/s and a maximum spot diameter of about 10 mm are estimated. Comparative values for the spot velocity were obtained from [26]. 

The cathode process provides the ion energies as shown in Figure 2 for 1450 A. An increase in the ion energy can be achieved by a bias voltage of 100 V and different pulse overlaps of 175 and 250 µs, which are shown in Figure 3. In Table 3, ion energies are summarized for the unbiased deposition (A) and the two depositions with different bias pulse durations at a 100 V bias voltage, whereas for (A) the energies are taken from the energy measurements (see Section 3.1.1). The energies for (B) and (C) are calculated considering the additional acceleration by the operating time of the applied bias voltage.

It can be seen that the mean ion energy is considerably influenced by bias acceleration. Due to the double-charged state of C^++^ ions, energies of up to 176 eV can be obtained, but due to their relatively small portion compared to the total number of ions, the mean energy is mainly affected by C^+^ ions. C^++^ ions increase the average energy by a maximum of 7 eV for case C.

### 3.2. Characterization of ta-C Coatings 

#### 3.2.1. Influence of Ion Energy and Heating

The effect of ion energy was studied using no bias and two different overlap times of 100 V bias voltage pulse (see Table 3 and Figure 3). The bias level of 100 V was chosen because, at this level, a maximum sp^3^ content is expected [12]. Table 4 shows the results of the coatings analysis of bias series A/B/C. Additionally, the results of coatings D and E, deposited with external heats of 120 and 140 °C, respectively, are provided. 

**Figure 4:**
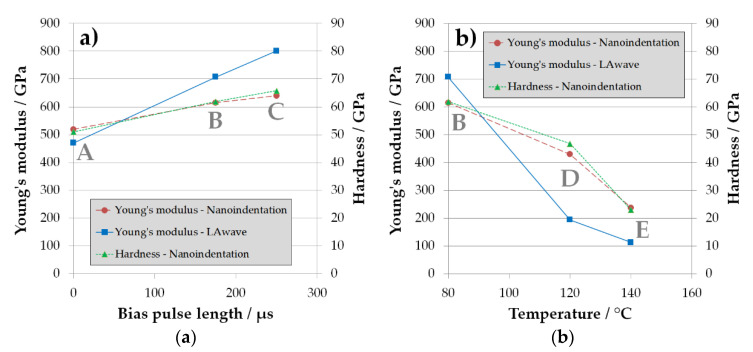
Dependence of the Young’s modulus (measured using two different techniques, nanoindentation and LAwave) and hardness on bias pulse length at 100 V, series A/B/C (**a**), and temperature, series B/D/E (**b**).

For the visualization of the effect of bias pulse length and temperature during deposition, the dependencies of series A/B/C and B/D/E on carbon coatings are shown in diagrams in Figure 4.

It is clear from Figure 4a that increasing the pulse length of the bias voltage leads to an increase in both ta-C hardness and Young’s modulus, similar to observations in the literature working with different bias voltage levels [27]. Obviously, not only the level but also the duration of applied bias voltage has a significant effect on hardness and Young’s modulus of the ta-C film. This can be explained because both the level and the duration of bias application in pulsed mode influence the mean energy of depositing carbon species (C^+^ and C^++^ ions) in the filtered arc plasma. According to Table 3, the mean energy starts from 31 eV (A) to 92 eV (B) and 117 eV (C), corresponding to the increasing bias pulse length. 

The hardness and Young’s modulus obtained from nanoindentation follow a clear trend with bias pulse length. The H/E ratio is fairly close to 0.1 and therefore consistent with the values published in the literature [28]. The results for the temperature series in Table 4 and Figure 4b show a clear decrease in hardness and Young’s modulus with temperature. This correlation confirms the results of other authors [16,29,30] and is explained by an increase in the sp^2^ proportion in the coatings at the expense of the diamond-like sp^3^-hybridised bonds. This relationship was also described theoretically by Schultrich [14]. 

There are some general discrepancies between Young’s moduli measured with LAwave and nanoindentation. The two methods have substantial differences. LAwave integrates properties over a small volume, including the full coating thickness, using strain amplitudes in the pm range and measuring in predominantly lateral direction. Contrary, information for nanoindentation comes from a comparable small volume around the indenter, using strain amplitudes in the nm range, while measuring in predominantly normal direction. Thus, differences between both methods are to be expected. For the bias series, the discrepancy of the different moduli is rather small, systematic, and pronounced only for harder coatings. For the temperature series, the discrepancies are opposite to the bias series, especially for softer coatings. Even more unexpected, the LAwave gives only values which are less than 50% of those from nanoindentation, as is the case for coating D. 

**Figure 5:**
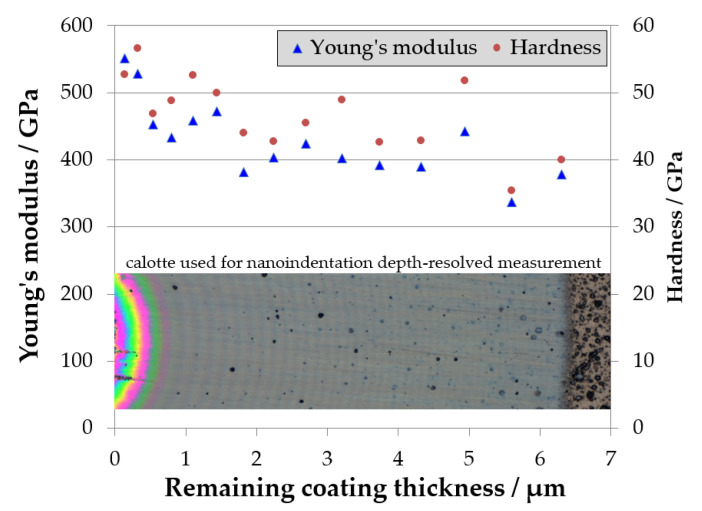
Young’s modulus and hardness values measured by nanoindentation in coating D. The values were obtained in different remaining coating thicknesses using a wedge-shaped area which was created by grinding a calotte into the layer. A microscope image of a calotte area is included.

In order to identify a possible gradient or inhomogeneities in the coating, depth-resolved nanoindentation was measured in coating D (120 °C) on a calotte being grinded by ball cratering. The results of these measurements are shown in Figure 5.

As can be seen in Figure 5, the coating has a hardness of about 50 GPa close to the film/substrate interface and shows a slight decrease in both hardness and Young’s modulus with increasing coating thickness. This might be explained by an additional heating due to self-heating by the kinetic energy of the deposited carbon ions. Starting from a 2 µm thickness, an equilibrium seems to be reached, and the coating is quite homogenous during further growth. The lower values near to the coating surface can be attributed to eventual surface effects and defects. 

However, the result of depth-resolved nanoindentation measurement does not help us to understand the discrepancy between the Young’s modulus from LAwave (194 GPa) and from nanoindentation (430 GPa). There is no indication of a low Young’s modulus using nanoindentation. The discussion on the hitherto unexplained discrepancy is continued at the end of this chapter and in Section 3.2.2.

For the structural investigation of all deposited coatings, Raman spectroscopy with the quantification of D- and G-peak position and I_D_/I_G_ ratio was employed. The chosen Lorentz/Breit–Wigner–Fano model could be well-fitted to the data, commonly reaching R^2^ > 0.998 for all single fits. 

**Figure 6:**
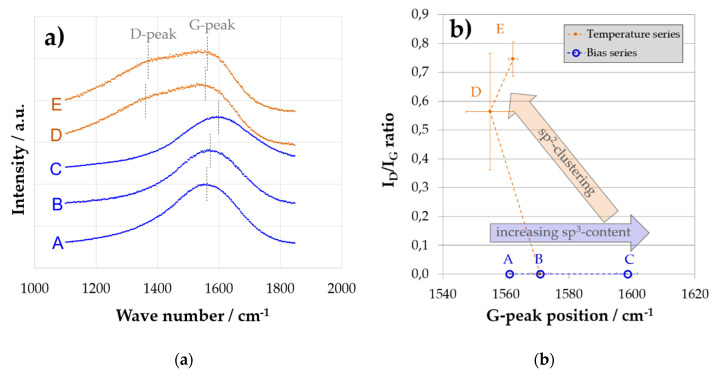
Raman plots (**a**) and results of the fitted Raman spectra are shown as I_D_/I_G_ peak height ratio over G peak position (**b**), visualizing independent influence of temperature and bias voltage on structural parameters. The blue arrow indicates direct correlation with an increased sp^3^ ratio, hardness, and Young’s modulus, where I_D_/I_G_ ≈ 0. The red arrow indicates an increase in graphitic nanoclusters with an emerging D peak.

For coatings A, B, and C, no carbon D peak can be found in the spectrum, indicating a fully amorphous carbon coating as obtained from the deposition process. In such a case, the G peak position correlates well with the sp^2^/sp^3^ ratio [19]. Consequently, the lowest peak position is found for coating A, with the lowest hardness and Young’s modulus. The highest peak position is found for coating C, correlating with the highest hardness and Young’s modulus (Figure 6). 

For coating D and E, a carbon D peak signal is evident. In that case, the original amorphous structure is lost, and nanoclustered graphitic sites have formed. Such behavior can be found whenever local rearrangement permits relaxing of the amorphous structure through increased energy input during or after deposition. Here, the elevated deposition temperature allows relaxation and nanoclustering of graphitic sites during deposition. For higher deposition temperatures, a pronounced D peak forms. As nanoclustering also affects the G peak position, no clear correlation between G peak position and hardness can be found where a D peak is present. 

As discussed previously, a pronounced discrepancy was found for moduli measured with LAwave and nanoindentation for coating D. The Raman results also show specific features for coating D, indicating a pronounced clustering of graphitic carbon, although coating D has only a slightly lower hardness than coating A (48 vs. 55 GPa). Consequently, this coating has similar mechanical properties during indentation while exhibiting a completely different structure of carbon atoms.

Combining the particularities found in the mechanical investigations and the Raman measurements, the following hypothesis is proposed: coating D is characterized by strong structural anisotropy due to an alignment of graphene planes in nanoclustered graphitic regions of the carbon film. Therefore, the in-plane properties (predominantly measured by LAwave) are different from those in a vertical load direction (measured by nanoindentation). To verify this hypothesis, additional investigations of coating D were made, as described in the following section.

#### 3.2.2. Structural Anisotropy in ta-C

First, coating D was subjected to comprehensive analysis by cross-section preparation in order to measure the in-plane properties with nanoindentation. In addition, a cross-section was prepared for TEM analysis to verify the hypothesis of aligned graphene lamellae in the graphitic nanoclusters by direct observation. 

**Figure 7:**
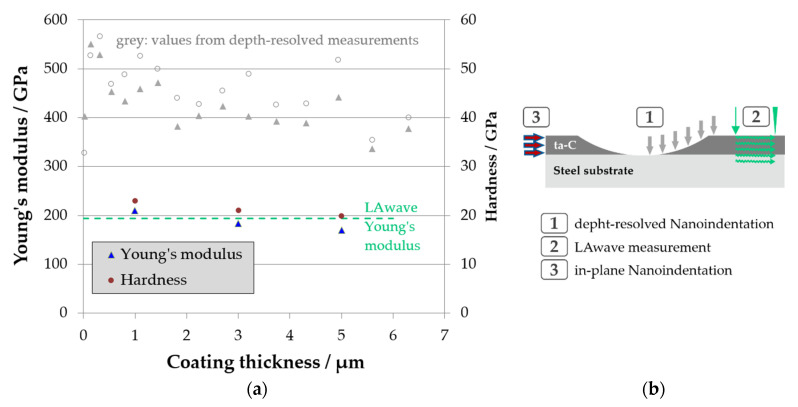
(**a**): in-plane Young’s modulus and hardness values measured by nanoindentation in coating D on a prepared cross-section. The values obtained by conventional, depth-resolved hardness measurement are included for illustration (grey symbols). The Young’s modulus value as measured by LAwave is added for comparison (dashed green line). (**b**): schematic illustration of the measuring directions of the different measuring techniques.

In order to measure the in-plane hardness and modulus of elasticity, a cross-section of the coated sample with a polished surface was prepared. Nanoindentation measurements were made near the interface, in the middle of the coating, and at the upper edge—i.e., at approx. 1, 3, and 5 µm away from the interface. The results are shown in Figure 7. In addition, Young’s moduli from depth-resolved nanoindentation (see also Figure 5) and LAwave were included for comparison. The results clearly show that both Young’s modulus and hardness in the in-plane direction are much lower than the values measured perpendicular to the coating plane. Furthermore, there is good agreement with the LAwave values of the Young’s modulus (E = 194 GPa), for which the in-plane properties also seem to be decisive.

The results strongly support the hypothesis of existing anisotropy in the layer. For further validation, the structure cross-section was investigated by TEM using high-resolution imaging and selected area diffraction (SAD) imaging.

**Figure 8:**
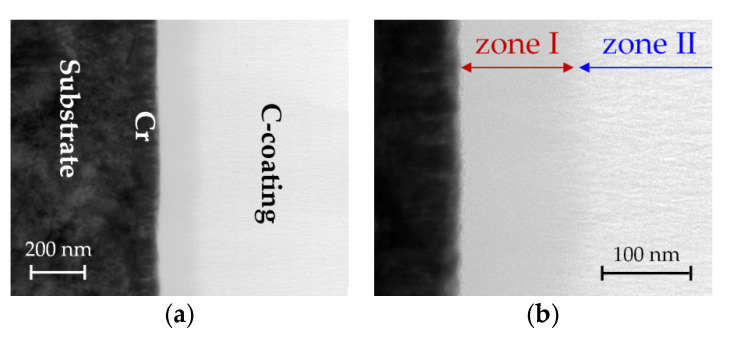
(**a**)TEM cross-section images of coating D. In particular, the higher magnification (**b**) shows a partition of the C-coating into a relatively structureless zone I and a nanostructured zone II for the main part of the coating.

**Figure 9:**
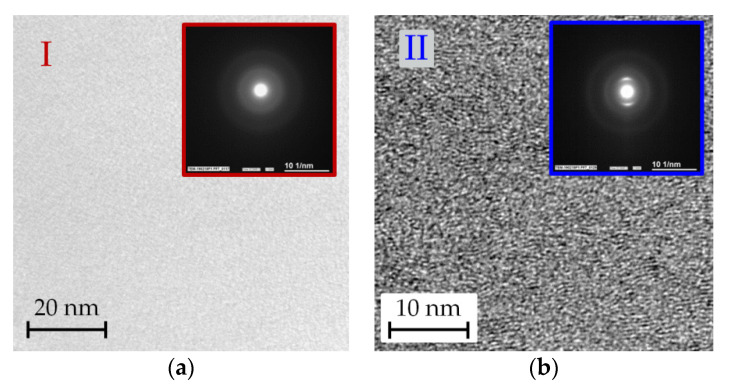
TEM cross-section images of coating D in high resolution and embedded SAD images. The assignment of zones I (**a**) and II (**b**) is shown in the image to the right in Figure 8.

Figure 8 shows the setup of coating D, comprising an approximately 100 nm-thick Cr adhesion layer and a carbon film. It is noticeable that the structure of the carbon is divided into an approximately 150 nm-thick zone I with an almost structureless appearance, and for the rest of the coating, zone II, which shows some nanostructural features. For deeper insight, high-resolution imaging and the local area diffraction of zones I and II were performed (see Figure 9).

Figure 8 shows that the approximately 150 nm-thick zone I of the ta-C layer at the interface is completely amorphous. The corresponding diffraction pattern in Figure 9 consists of uniform diffuse rings, which are typical for the structure of amorphous carbon. The analysis of zone II (right picture), on the other hand, shows larger lamellar zones, which are clearly aligned in the direction of the coating normal axis. In the diffraction image, local intensity maxima can be seen, which point to crystalline areas and indicate texturing. From the position of the maxima, the distance between the network planes can be estimated at about 0.3 nm, which corresponds to the distance between the graphene planes in crystalline graphite (0.335 nm). It becomes clear that after a growth of about 150 nm, amorphous ta-C starts the formation of vertically oriented graphene planes, which extend over areas in the order of 10 nm. These regions are embedded in an amorphous carbon structure.

With these observations from the TEM analysis, the findings from the mechanical analyses with the discrepancy between the in-plane mechanical properties and those in the direction of the layer normal can be explained. The reason for this is a structure-related anisotropy in the carbon coating. The graphene lamellae within smaller graphitic regions are preferably aligned in the direction of growth. It can be assumed that the arrangement of the lamellae is parallel to the normal, but otherwise arbitrarily oriented—this structure is known as transverse isotropy. The effect is analogous to the alignment of the graphene lamellae in carbon fibers, where it also provides a strong property anisotropy—i.e., a much higher Young’s modulus in the direction of the fiber [31].

## 4. Conclusions

Tetrahedral amorphous carbon coatings were deposited with plasma-filtered laser-arc technology while studying the influence of bias voltage characteristics and deposition temperature on the plasma and coating properties. A fully ionized carbon plasma with a significant amount of double-charged ions of up to 10% was found. The peak energy for the discharge currents of 1100–1450 A was in the range of 15–19 eV and the average energy was approximately 31 eV in all cases. At the selected discharge current, a maximum arc spot extension of approximately 10 mm was obtained when the maximum current was reached. A maximum arc spot velocity of 40 m/s was observed in the current rise phase.

The properties of the ta-C coatings deposited by filtered laser-arc deposition can be considerably altered by the bias voltage and by additional substrate heating. An increase in the pulse overlap of bias and arc-current pulse led to an increase in the hardness and Young’s modulus. The use of external heating, on the other hand, led to a noticeable reduction in the hardness and Young’s modulus. Both effects were attributed to the influence of the deposition parameters on the global sp^3^ content in the coatings.

External heating during the Laser-arc deposition of ta-C could cause the formation of strong transverse isotropy in a medium temperature range of about 120 °C. The reason for this lies in the formation of graphitic regions and the orientation of the graphene lamellae in the film normal direction. As a result, the coating exhibited a high level of hardness under a vertical load. At the same time, the coatings were elastic and relatively soft in-plane. This type of coating is of much interest, as it combines superhardness with a high deformability. In highly loaded contacts, in particular, this coating could have superior properties to conventional ta-C coatings. At higher temperatures (approximately > 140 °C), the effect is much less pronounced and the coatings become comparatively soft again, as is known from sputtered a-C [32].

Our investigations have shown that the mechanical and structural properties of ta-C coatings are highly dependent on the bias voltage, pulse length, and substrate temperature during Laser-Arc deposition. Using special deposition parameter windows, highly anisotropic amorphous carbon coatings with novel, superior properties can be achieved. Thus, with a full understanding and full control of the complex deposition process, reliable and tailored ta-C coatings can be achieved for various applications on components and tools.

## Figures and Tables

**Table 1:** Parameters used to synthesize different variants of laser-arc carbon coatings.

Coating Designation	Bias Voltage (V)	Bias Pulse Duration (µs)	Heating Temperature (°C)
A	0	0	80 ^1^
B	100	175	80 ^1^
C	100	250	80 ^1^
D	100	175	120
E	100	175	140

^1^ Equilibrium temperature (no external heating).

**Table 2:** Mean energy of single- and double-charged carbon ions at 1100–1450 A arc current.

Current (A)	Mean Energy		C^++^/C^+^ Ratio
	C^+^ (eV)	C^++^ (eV)	
1100	30.8	16.7	4.3
1300	31.3	18.3	7.5
1450	32.6	19.6	10.4

**Table 3:** Mean energy of the carbon ions for coatings A, B, and C.

Designation	Bias Voltage (V)	Bias Pulse Duration (µs)	Mean Energy		
			C^+^ (eV)	C^++^ (eV)	Total (eV)
A	0	0	33	20	31
B	100	175	87	129	92
C	100	250	111	176	117

**Table 4:** Measured coating properties for carbon coatings A–E.

Designation	LAwave Young’s Modulus (GPa)	Nanoindentation Young’s Modulus (GPa)	Nanoindentation Hardness (GPa)	Coating Thickness (µm)
A	470	519	51	4.0
B	707	615	62	2.0
C	801	640	66	1.1
D	194	430	47	6.2
E	113	238	23	3.2

## Data Availability

Data sharing is not applicable to this article.
